# The impact of body position on neurofluid dynamics: present insights and advancements in imaging

**DOI:** 10.3389/fnagi.2024.1454282

**Published:** 2024-11-08

**Authors:** Marco Muccio, Zhe Sun, David Chu, Brianna E. Damadian, Lawrence Minkoff, Luciano Bonanni, Yulin Ge

**Affiliations:** ^1^Department of Radiology, NYU Grossman School of Medicine, New York, NY, United States; ^2^FONAR Corporation, Melville, NY, United States; ^3^Department of Radiology, Northwell Health-Lenox Hill Hospital, New York, NY, United States

**Keywords:** neurofluids dynamics, aging, neurodegeneration, body position effects, neuroimaging, phase contrast MRI

## Abstract

The intricate neurofluid dynamics and balance is essential in preserving the structural and functional integrity of the brain. Key among these forces are: hemodynamics, such as heartbeat-driven arterial and venous blood flow, and hydrodynamics, such as cerebrospinal fluid (CSF) circulation. The delicate interplay between these dynamics is crucial for maintaining optimal homeostasis within the brain. Currently, the widely accepted framework for understanding brain functions is the Monro-Kellie’s doctrine, which posits a constant sum of intracranial CSF, blood flow and brain tissue volumes. However, in recent decades, there has been a growing interest in exploring the dynamic interplay between these elements and the impact of external factors, such as daily changes in body position. CSF circulation in particular plays a crucial role in the context of neurodegeneration and dementia, since its dysfunction has been associated with impaired clearance mechanisms and accumulation of toxic substances. Despite the implementation of various invasive and non-invasive imaging techniques to investigate the intracranial hemodynamic or hydrodynamic properties, a comprehensive understanding of how all these elements interact and are influenced by body position remains wanted. Establishing a comprehensive overview of this topic is therefore crucial and could pave the way for alternative care approaches. In this review, we aim to summarize the existing understanding of intracranial hemodynamic and hydrodynamic properties, fundamental for brain homeostasis, along with factors known to influence their equilibrium. Special attention will be devoted to elucidating the effects of body position shifts, given their significance and remaining ambiguities. Furthermore, we will explore recent advancements in imaging techniques utilized for real time and non-invasive measurements of dynamic body fluid properties *in-vivo*.

## 1 Introduction

The human cranium serves as an enclosed space where the structural and functional properties of the brain are susceptible to external influences such as postural and gravity changes, cardiac rhythm and respiration, as well as intracranial pathologies such as tissue edema, tumor, trauma and neurodegeneration. Two primary dynamic fluid systems – arterial and venous blood, along with cerebrospinal fluid (CSF) – play integral roles in maintaining central nervous system (CNS) homeostasis and respond adeptly to external influences.

Disruptions in hemodynamics and CSF hydrodynamics have been observed in various neurological conditions. For example, reduction in cerebral blood supply has been observed to correlate with transient ischemic attack (TIA) (Sui et al., 2023; Prabhakaran et al., 2011), white matter hyperintensity (WMH) (Huang et al., 2021; Alosco et al., 2013), and stroke (Demeestere et al., 2020). In addition, impaired CSF production or absorption could lead to misfolded protein accumulation in the brain, raising toxicity and consequently trigger neurodegeneration. Recent studies have in fact emphasized that CSF circulation serves not only as providing mechanical and nutrient support to the brain but also regulating the extracellular environment and removing waste byproducts of neuronal metabolism. Reduced waste clearance could lead to disturbances in intracranial pressure (ICP), accumulation of toxic substances and impaired glymphatic system as observed in aging (Kress et al., 2014), sleep issues (Xie et al., 2013) and conditions such as hydrocephalus and normal pressure hydrocephalus (NPH) (Linninger et al., 2007; Bateman and Brown, 2012; Symss and Oi, 2013). It has been hypothesized that such impairment in circulation could contribute to the accumulation of amyloid-beta and tau proteins in the brain, exacerbating neuronal damage and cognitive decline (Iliff et al., 2014; Wang et al., 2017).

Age stands as the foremost significant factor in Alzheimer’s disease (AD) and AD-related dementia (ADRD). As individuals age, typical processes often include a reduction in sleep duration and less time spent in the lying down position (supine), therefore increasing the total time spent in the upright position. Nevertheless, the impacts of ubiquitous factors on neurofluid dynamics from daily changes of body position, experienced by everyone, remain relatively unknown.

In recent years, there has been a growing interest in understanding the effects of body position on human body fluid physiology. Evidence suggests that changes in posture induce alterations in various physiological processes such as blood and CSF circulation, deceleration of heart rate, reduction in blood pressure, and adaptation of the autonomic nervous system (Sagirov et al., 2023). As such, it is reasonable to hypothesize that this seemingly simple factor –upright or supine body position– may hold significant implications for optimizing and maintaining brain health.

Previous investigations, whether at the preclinical or clinical level, have mostly focused on exploring hemodynamic or hydrodynamic changes in isolation, despite these two factors have been shown to significantly influence each other (Figley and Stroman, 2007; Beltran et al., 2023). Therefore, the goal of this work is to present a comprehensive picture of the impact of body position on both hemodynamic and hydrodynamic aspects. Additionally, we will explore recent advancements in neuroimaging technologies, allowing for real-time, *in-vivo* monitoring of blood and CSF flow in humans and their application in age-related neurodegenerative diseases.

## 2 Hemodynamics and hydrodynamics of CNS

### 2.1 Hemodynamics

The human body’s hemodynamic properties consist of two distinctive cardiovascular systems: the arterial system, delivering oxygenated blood and nutrients to organs and tissues; and the venous system, returning deoxygenated blood to the heart and lungs for reoxygenation and recirculation. This blood circulation is driven by the cardiac flow, significantly impacting cerebral perfusion. A slower resting heart rate (HR) (<75 bpm) is often indicative of a healthier cardiovascular system compared to faster rates (Fox et al., 2007; Palatini et al., 1997). This may be attributed to the fact that higher HR can lead to increased blood pressure, greater cardiac output (Miyai et al., 2002; Palatini and Julius, 1997) and increased vascular wall shear pressure (Taylor et al., 2002; Yap et al., 2012), potentially compromising cardiovascular integrity especially at long timescales (Topper and Gimbrone, 1999). In addition, HR variability (HRV) in an individual could be used as an indicator of cardiovascular and cerebrovascular health (Shaffer et al., 2014), with lower HRV associated with increased mortality risk (Tsuji et al., 1994; Kleiger et al., 1987) and disease susceptibility (Thayer et al., 2012; Carney et al., 2005). Thus, maintaining efficient and adaptive circulation mechanisms is essential for optimal health.

The human brain typically receives 15–20% of the cardiac output, around 50 mL/100 g/min (Vavilala et al., 2002), primarily from the bilateral internal carotid arteries (ICAs) (72%) and the bilateral vertebral arteries (VAs) (28%) (Zarrinkoob et al., 2015). Cerebral blood flow (CBF) is meticulously regulated to meet the brain’s metabolic and oxygen needs through cerebral autoregulation (Silverman and Petersen, 2024), ensuring optimal function. It is widely believed that total CBF is higher in females than in males (Vernooij et al., 2008; Tarumi et al., 2014) and decreases with age (Tarumi et al., 2014; Amin-Hanjani et al., 2015). However, these findings remain contentious, as some studies reported no sex-related effects (Amin-Hanjani et al., 2015; Buijs et al., 1998). Additionally, alongside common vascular tortuosity changes with age (Sun et al., 2022; Li et al., 2024), other vascular pathologies, like arteriosclerosis and atherosclerosis, can disrupt cerebral perfusion and autoregulation, even in individuals without clinical symptoms (Han et al., 2019; Hecht et al., 2021; Kaczmarz et al., 2021).

Cerebral blood flow coupling, vital for brain homeostasis, involves a complex interplay between blood and neuronal cell dynamics, regulated by neurovascular coupling (NVC), a crucial brain phenomenon that can be assessed with blood-oxygen-level-dependent (BOLD) MRI (Kim and Ogawa, 2012; Ogawa et al., 1990; Attwell and Iadecola, 2002). The NVC is dynamically regulated by components like astrocytes and the vascular endothelium (Thakore et al., 2021; Pellerin et al., 2007). Disruptions in vasomotor function or NVC can impact vascular modulation and its downstream fluid dynamics, potentially leading to neurodegeneration, as observed even in healthy aging (Wilson and Matschinsky, 2020).

**FIGURE 1:**
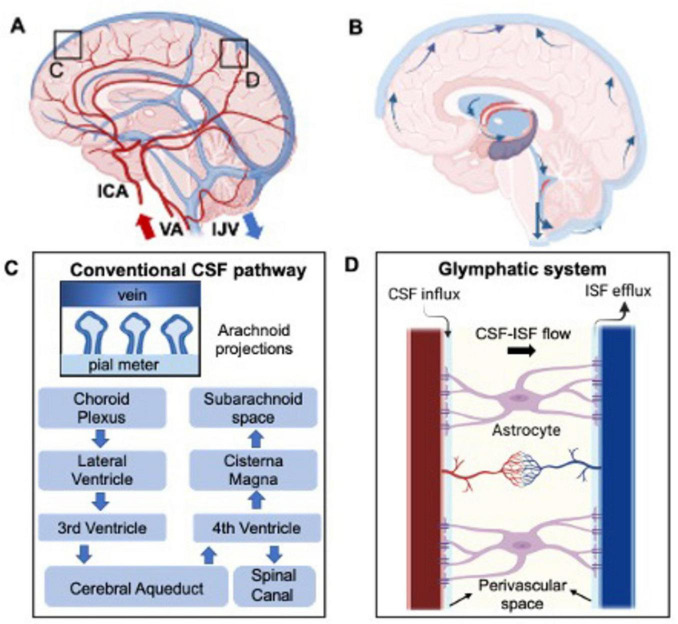
Depiction of the healthy hemodynamic **(A)** and hydrodynamic system **(B)** concerning cerebral blood and CSF flow, respectively. **(C)** Represents in more details the CSF flow in the brain and the hypothesized clearance pathway into the venous blood flow through the arachnoid projections. This, named the glymphatic system, is even more detailed in part **(D)**, where CSF-interstitial fluid (ISF) exchange is highlighted.

Of equal importance is the cerebral venous system, responsible for removing deoxygenated blood and, as recently suggested, clearing waste products resulting from neuronal activity. This system consists of non-contractile vessels, including superficial and deep veins and the major dural sinuses, facilitating drainage through the internal jugular veins (IJVs) toward the heart and lungs for oxygenation (Shapiro et al., 2023; Valdueza et al., 1996). Unlike arteries, IJVs can partially collapse (change in diameter) in response to external pressure changes (Schaller, 2004). Although often overshadowed by arterial flow, understanding of the venous drainage is crucial for its role in maintaining brain functionality. These arterial and venous hemodynamic properties are illustrated in Figure 1A.

### 2.2 Hydrodynamics

Cerebrospinal fluid dynamics are fundamental for maintaining brain homeostasis by facilitating fluid circulation. The choroid plexus (ChP), located in brain’s ventricular system, produces 500 mL per day with 3–4 times turn over in young adults (Dandy, 1919; MacAulay et al., 2022; Huff et al., 2023). Here, plasma is filtered through the epithelial cells of the ChP (Johanson et al., 2008; Brown et al., 2004). The CSF then circulates from the lateral ventricles to the third and fourth ventricles via the cerebral aqueduct (or aqueduct of Sylvius). From this point, the CSF drains into the spinal subarachnoid space as well as reaching the cisterna magna at the craniocervical junction and draining into the cerebral subarachnoid space (Figure 1B). CSF is then filtered into the cerebral venous blood through small protrusions called arachnoid villi or granulations. These structures, mainly observed in major venous sinuses such as the superior sagittal sinus, increase the contact area between CSF and the venous blood (Khasawneh et al., 2018; Pollay, 2010; Upton and Weller, 1985) therefore increasing the transport of waste out of the surrounding brain tissue (Figure 1C). Additional absorption sites include the bulbar nerve, lymphatic nodes, and ventricular walls (Miyajima and Arai, 2015; Chen et al., 2015). The rate of CSF absorption is closely related to the CSF flow and ICP, which creates a pressure gradient necessary for filtering out the toxic byproducts of brain activity.

The recent identified glymphatic system is believed to mainly involve CSF circulation in periarterial and perivenous CSF spaces connected by interstitial fluid (ISF), where toxic substances produced by cells like neurons and glial cells primarily accumulate (Ringstad and Eide, 2024; Figure 1D). CSF is thought to circulate in a pulsatile manner, driven by brain and spinal cord movements associated with cardiac pulsation (Lagana et al., 2022b; Baselli et al., 2022), respiration (Lagana et al., 2022a; Dreha-Kulaczewski et al., 2015), and cellular motile cilia (Kumar et al., 2021). Arterial and venous flow, including pressure gradients and intracranial pressure (ICP) are proposed as the main drivers of CSF circulation (Alperin, 2020). These interactions between brain hemodynamics and hydrodynamics are crucial for maintaining brain homeostasis and optimal functioning especially in response to external stimuli.

## 3 Body position influences

Several imaging studies have explored how body position affects brain fluid dynamics and intracranial pressure, primarily using animal models because the standard clinical MRI scanners do not allow for upright imaging. These studies have shown that changes in body position alter CSF flow and overall intracranial pressure. Klarica et al. (2014) demonstrated in cats that CSF pressures in the lateral ventricles increases, and lumbar subarachnoid space pressure decreases, with increasing head inclination (0–90 degrees). Moreover, they reported opposite trends when the head and body were tilted downward (225 and 270 degrees) (Klarica et al., 2014). Similar findings have been supported by other studies (Klarica et al., 2022; Lee et al., 2015; Kuzman et al., 2012; Carlson et al., 2003) with recent interest in understanding body position’s influence on blood and CSF compliance (Podgorsak et al., 2023).

**FIGURE 2:**
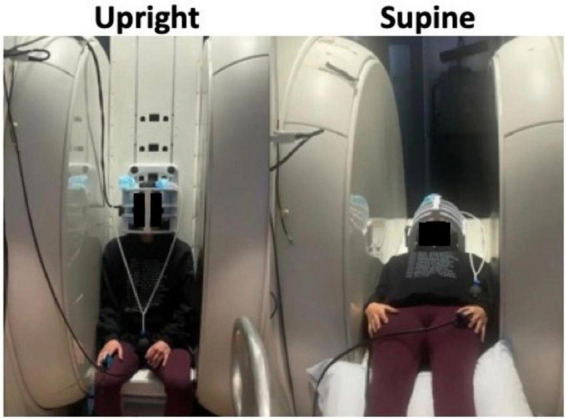
Image of subject scanned in an upright MRI scanner showing how it is possible to shift the position between upright **(left)** and supine **(right)** within the MRI scanner itself. Adapted with permission from Muccio et al. (2021), *Fluids Barriers CNS*.

In addition to circulation effects, rodent studies have shown that different body positions affect brain waste clearance. Lee et al. (2015) found that convective flux from CSF to interstitial space and radiotracer clearance were greater in supine (or lateral) positions compared to upright (or prone) in anesthetized rodents. Human studies have been mainly restricted to mathematical simulations (Lakin et al., 2003) to calculate CSF flow changes in healthy individuals (Muccio et al., 2021; Alperin et al., 2005a) due to the hardware limitations and availability of upright MRI scanners. Some recent imaging studies, however, have investigated the body position effects in patients with hydrocephalus and/or Chiari malformations (Farahmand et al., 2015; Poca et al., 2006). This section will discuss the impacts of postural changes on hemodynamics and hydrodynamics in humans, particularly using MRI scanners which allow imaging in both upright and supine positions (Figure 2).

### 3.1 Hemodynamic changes

Changes in heart rate (HR) following a shift in body position indicate cardiovascular responsiveness and adaptability. Moving from supine to upright positions typically leads to increased HR, HRV (Muccio et al., 2021; Tikkakoski et al., 2013; Watanabe et al., 2007; Smith et al., 1994), blood pressure and other chemical changes (Tulen et al., 1999). This compensatory response ensures sufficient blood supply to vital organs, particularly the brain, which is the farthest vital organ from the body’s center of gravity. Known as the orthostatic or postural reflex, this process helps prevent orthostatic hypotension, more common in older individuals due to their reduced vascular reactivity (Smith et al., 1994; Ricci et al., 2015; James and Potter, 1999). Recent studies propose that HR changes from supine to upright positions could predict prognosis in chronic heart failure patients. Higher HR increases due to postural changes correlate with lower risk of heart failure, reflecting augmented vasomotor modulatory capability (Maeder et al., 2016). These findings underscore the importance of a dynamic cardiovascular system able to support fluid dynamics shifts as response to postural changes. Furthermore, HR variations with body position significantly affect CBF and intracranial perfusion.

Studies have explored the link between head tilt and CBF in humans. While increasing head angulation up to 90 degrees (upright) typically reduces cerebral perfusion immediately after the position shift; some studies showed no significant differences at lower angles (<30 degrees) (Kose and Hatipoglu, 2012). In the supine position, cerebral arteries can more effectively dilate and constrict in response to blood flow changes compared to the upright position (Favre et al., 2020). These differences in blood circulation between the supine and upright postures are influenced by gravity’s effects on fluid dynamics, as explained by Bernoulli’s principle of conservation of energy in fluids. In the supine position, the cardiac system is uniformly affected by gravity, whereas in the upright position a hydrostatic gradient forms, leading to higher pressure in the feet and lower pressure in the brain (Hinghofer-Szalkay, 2011). To counteract these gradients, blood vessels must adjust by dilating or constricting in specific areas to ensure consistent blood perfusion to vital organs like the brain (Hinghofer-Szalkay, 2011). On the venous side, in the supine position, most cerebral blood flow exits via bilateral IJVs but, in the upright position, gravity causes an increase in surrounding pressure and consequent collapse of these veins, rerouting cerebral venous blood through smaller cervical veins known as the venous plexus, necessary to maintain optimal intracranial pressure regulation (Kosugi et al., 2020; Holmlund et al., 2018; Valdueza et al., 2000). Additionally, the CNS may directly influence hemodynamic adaptation to postural changes via the sympathetic nervous system, detecting initial blood pressure shifts through baroreceptors and subsequently increasing HR and blood flow (Fisher and Paton, 2012; Joyner et al., 2010; ter Laan et al., 2013).

Understanding dynamic cerebral blood flow variations with body position is crucial for grasping brain autoregulation complexities and may offer insights into cardiovascular health and early signs of vascular diseases.

### 3.2 Hydrodynamic changes

**FIGURE 3:**
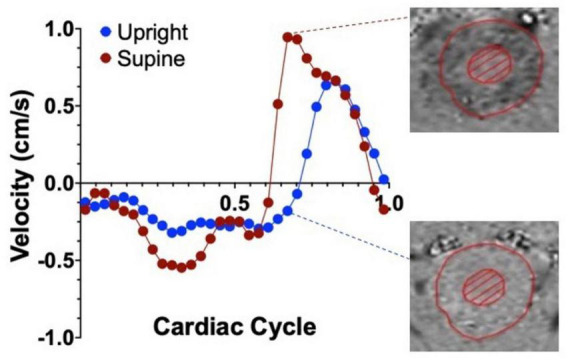
Representative graph of the average cervical CSF velocities within a single cardiac cycle, in the upright (blue dots) and supine (red dots) positions in healthy controls. Positive values represent CSF flowing in the caudal direction and negative velocities represent flow in the cranial direction. Notice the velocity difference between the two positions, highlighted by the phase contrast images on the right. Adapted with permission from Muccio et al. (2021), *Fluids Barriers CNS*.

The effects of body position on cranial CSF flow are often underestimated despite their crucial role in maintaining brain homeostasis. A preliminary search for “CSF flow and body position” in PubMed yielded only 32 entries, mainly focusing on mathematical modeling or shunt valve mechanics. Among these, Muccio et al. (2021) demonstrated that CSF flow at the cervical level is over 50% greater in the supine position compared to upright, indicating increased fluid exchange between the spinal canal and the cranium in the supine position (Muccio et al., 2021; Figure 3). Previous MRI studies also support this decrease in CSF volume exchange in the upright versus sitting position (Alperin et al., 2005a; Alperin et al., 2021; Alperin et al., 2005b). Factors influencing these postural changes in CSF flow include spinal decompression and cranial/spinal compliance. Notably, significant spinal cord decompression occurs in the supine position, affecting CSF flow dynamics following basic fluid dynamics principles (Kuwazawa et al., 2006; Cadotte et al., 2015). In the horizontal position, the cranial compartment contributes 37% and the spinal compartment 63% to the total craniospinal compliance. Conversely, in the upright position, these values nearly reverse, with the cranial compartment contributing 66% and the spinal compartment 34% (Magnaes, 1989). Physiologically, greater CSF velocity but lower stroke volume (or volume displaced bidirectionally) is observed in the narrowing of the spinal canal (Beltran et al., 2023), potentially influenced by factors like age and sex (Beltran et al., 2023; Yanase et al., 2006).

Despite extensive literature on CSF dynamics, research specifically focusing on the effects of body posture on CSF production or secretion is lacking, largely due to challenge in non-invasive measurement of these CSF properties, complicated by the limited spatial resolutions of upright imaging technologies. On the other hand, studies on astronauts exposed to microgravity have demonstrated changes in CSF hydrodynamics, supported by a substantial increase in CSF production post-flight (Kramer et al., 2020; Kramer et al., 2015), along with changes in CSF volume due to microgravity exposure (Roberts and Petersen, 2019; Roberts et al., 2017). Considering this microgravity or weightless environment as extreme of supine position hypotension, it is speculated that CSF production might increase in supine compared to the upright posture, though not to the same extent as in microgravity.

Body posture also influences the efficiency of glymphatic pathways, crucial for distributing nutrients and removing brain waste. This system is affected by sleep, anesthesia, respiration, and arterial pulsation, driving glymphatic flux by enlarging the interstitial fluid (ISF) space, lowering resistance to the perivascular inflow, and enhancing CSF-ISF exchange (Xie et al., 2013; Iliff et al., 2013; Schneider et al., 1998). Recent rodent studies using contrast-enhanced MRI and optical imaging suggest faster clearance of contrast agents in lateral resting positions, indicating more efficient glymphatic transport (Lee et al., 2015). However, further research, especially in humans, is needed to validate these findings, especially considering confounding factors linked to anesthetics use in animals and awaiting clinical validation.

Understanding how external factors, such as body position, affect CSF circulation is pivotal for comprehending conditions where this intricate system is compromised and potentially even predict the trajectory of neurodegeneration in disease as well as in healthy aging.

### 3.3 CBF-CSF coupling changes

To maintain stable ICP, hemodynamics and hydrodynamical changes must synchronize in timing and magnitude. Recent orthostatic MRI studies report interactions between blood and CSF flows (Figley and Stroman, 2007; Beltran et al., 2023), but none have directly investigated the body position shifts’ effect on blood-CSF flow coupling.

Current understanding suggests that standing (or upright position) causes venous blood pooling in the lower limbs and the collapse of the IJVs, reducing cranial blood outflow and briefly increasing ICP, which is counterbalanced by increased CSF circulation caudally, or out of the intracranial space. Conversely, moving from a standing to supine opens the IJVs, increasing venous outflow and temporarily lowering the ICP. This is countered by increased cranial CSF flow cranially, into the intracranial space, and larger CSF volume exchanged between the cranial and spinal space (Muccio et al., 2021). Studies also showed that arterial blood flow is greater in the supine position compared to upright, balanced by greater venous outflow (Alperin et al., 2015) and that the bidirectional CSF flow between the spinal canal and intracranial space follows arterial and venous blood flow differentials. This highlights the intricate interactions between hemodynamic and hydrodynamic properties, where changes in one affect the other, maintaining optimal ICP through a delicate compensatory mechanism. Understanding these dynamics is crucial for appreciating brain autoregulatory mechanisms especially in response to external forces and stimuli.

**FIGURE 4:**
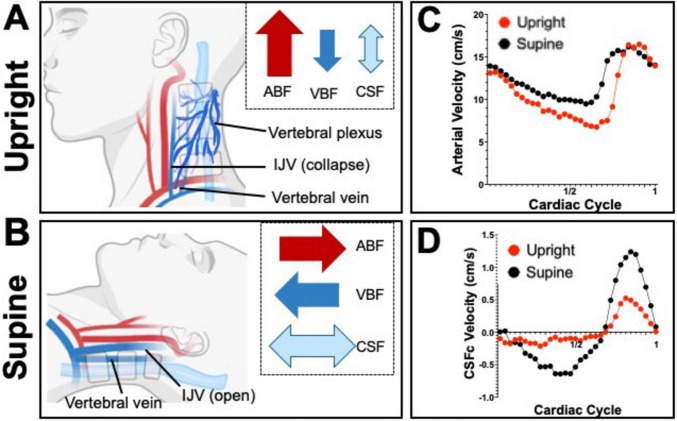
Schematics depicting the dynamic hemodynamic and hydrodynamic differences due to body position in upright **(A)** and supine **(B)**. Notice how, especially in CSFc, exchange between the spinal and cranial spaces is greater in the supine position and it is mimicked by an increased in venous blood flow (VBF) whilst arterial blood flow (ABF) barely increases. The proportion of the arrows’ size is exaggerated to better highlight the differences in flows between the two body positions. Graphs **(C,D)** show flow velocity differences between supine (black dots) and upright (red dots), over a single cardiac cycle, for arterial blood and cervical CSF (CSFc), respectively.

Cerebrospinal fluid production, on the other hand, is a continuous, dynamic process finely tuned with CSF absorption rates to maintain the appropriate volumes of CSF necessary for cushioning, support, and waste removal in the CNS. Ongoing research enhances our understanding of neurophysiology and aids in developing diagnostic and therapeutic strategies for conditions affecting vascular health, CSF dynamics and ICP regulation. There is a significant gap in understanding how postural changes affect CSF absorption and waste clearance, with current insights largely speculative and based on indirect assessment of CSF volumes or flow changes. Further clinical studies are needed to directly observe the effects of body position on CSF and neuronal waste clearance. Figure 4 further illustrates the comprehensive changes in hemodynamic and hydrodynamic flows due to body position shifts.

## 4 Other factors

Earlier investigations of the modulation of hemodynamic and hydrodynamic characteristics of the brain mainly concentrated on influencing variables such as exercise, respiration rates and sleep.

### 4.1 Vascular

Cerebrospinal fluid hydrodynamics are significantly influenced by various physiological forces, particularly of vascular nature. The influence of high frequency (∼1 Hz) and low-frequency oscillations (LFOs) have been well established and support the theory that the cardiac pulsations have strong effects on CSF flow, following the Monro-Kellie doctrine (Lagana et al., 2022b; Strik et al., 2002). In particular, LFOs (0.01 to 0.1 Hz) have in fact been demonstrated to be primary drivers of CSF flow especially during sleep in both animals (Bojarskaite et al., 2023) and humans (Fultz et al., 2019; Yang et al., 2022). Studies indicate that LFOs facilitate the clearance of waste from the brain, underscoring their importance in maintaining neurological health (Bojarskaite et al., 2023).

These physiological forces exhibit coupling and cross-frequency interactions that collectively shape CSF flow. For instance, such vascular influences have been observed to be closely coupled with the influence that respiration, whether free or forced, has on the CSF properties (Vijayakrishnan Nair et al., 2022; Yildiz et al., 2017). This interaction is crucial as it demonstrates how mechanical forces acting on the brain can integrate to enhance or restrict CSF circulation.

### 4.2 Respiration

Respiratory activities, such as inhalation and exhalation, significantly affect CSF flow dynamics within the spinal canal (Dreha-Kulaczewski et al., 2018; Delaidelli and Moiraghi, 2017). Dreha-Kulaczewski et al. (2017) demonstrated that forced breathing increases venous efflux from the cranium and enhances cranial CSF flow during inhalation, while decreasing venous blood flow and increasing CSF efflux into the spinal canal during exhalation. Subsequent studies with healthy volunteers confirmed these findings, noting decreased flow rates in the IJVs, superior sagittal sinus (SSS) and ICAs during forced deep breathing, along with increased CSF flow rates and decreased peak caudal CSF flow (Lagana et al., 2022b; Lagana et al., 2022a; Kollmeier et al., 2022). Variations in cervical CSF flow were observed within a single respiratory cycle, with cranial flow during expiration (Yildiz et al., 2017; Dreha-Kulaczewski et al., 2018), likely attributable to thoracic pressure changes induced by diaphragm movement, especially during rapid expiration events like coughing or sneezing.

### 4.3 Physical exercise

Exercise and overall fitness have been shown to significantly influence hemodynamic and hydrodynamic mechanisms (Smith and Ainslie, 2017; Ogoh and Ainslie, 2009). Exercising decreases blood oxygenation levels, especially during high-intensity sessions (Mekari et al., 2015), which is offset by an increased cerebral perfusion (Querido and Sheel, 2007; Ogoh et al., 2005; Heckmann et al., 2003; Brys et al., 2003; Ide et al., 1999; Jorgensen et al., 1992). Moreover, recent studies suggest that active individuals have greater intracranial CSF flow and absorption compared to less active controls (Edsbagge et al., 2004). In addition, active individuals also have greater intracranial CSF egress into venous blood compared to sedentary individuals. But when exercise hours and efforts are increased in sedentary people, an improvement of the CSF pathways system is observed (Miyazaki et al., 2024). Exercise also has effects on the coupling of blood and CSF. An increase in arterial and venous blood flow in the neck vessels, likely a result of elevated HR and blood pressure, correlates with decreased CSF stroke volume in the aqueduct of Sylvius (Tarumi et al., 2021).

### 4.4 Sleep

During sleep, the brain experiences considerable hydrodynamic alterations, impacting cognitive function and overall health. Quality sleep is crucial for memory consolidation (Peigneux et al., 2004; Sutherland and McNaughton, 2000), task performance and mood regulation. Neuroimaging studies reveal biodynamic brain changes during different sleep stages, including changes in regional CBF (Peigneux et al., 2004; Klingelhofer et al., 1995), regional metabolism (Nofzinger et al., 2002), functional connectivity (Banks et al., 2020; El-Baba et al., 2019; Samann et al., 2011), and reduced cellular metabolisms following sleep deprivation (Thomas et al., 2000). Sleep affects brain fluid dynamics, particularly CSF movement, with the glymphatic system being active during sleep to eliminate neurotoxic byproducts accumulated during periods of wakefulness (Smets et al., 2023; Chong et al., 2022). Even a single night of sleep deprivation has been observed to reduce CSF clearance (Eide et al., 2022; Shokri-Kojori et al., 2018).

Advanced neuroimaging techniques are currently used to study intracranial CSF and blood flow characteristics (Desseilles et al., 2008), aiding in understanding sleep-related changes and their distinction from body-position-induced alterations. Further research in both healthy individuals and those patients with sleep-related disorders (e.g., insomnia, sleep apnea, restless leg syndrome) is needed to distinguish the changes related to sleep and the ones induced by body position.

Research in this area often employs advanced imaging techniques, such as MRI, to investigate such characteristics of intracranial CSF and blood flow. These imaging techniques have provided a way to non-invasively and accurately measure such flow properties in the context of body position shifts.

## 5 MRI flow measuring techniques

Many direct and indirect techniques have been used to investigate the brain fluid dynamic properties and the factors affecting them. This report focuses on major non-invasive imaging methods. Clinically, phase contrast MRI (PC-MRI), a fast and robust technique established since the 1980s (Bryant et al., 1984; Moran, 1982; Pelc et al., 1991), is often used to measure blood and CSF flow.

### 5.1 Regular PC-MRI

**FIGURE 5:**
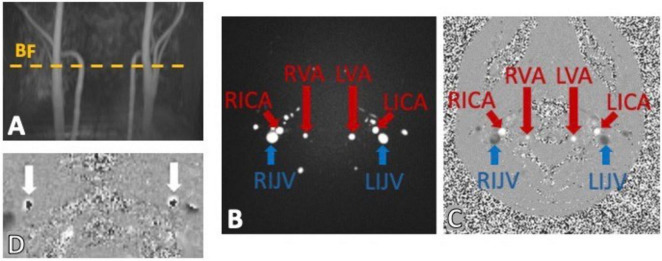
Example of phase contrast MRI (PC-MRI) sequence output using a time-of-flight (TOF) to place the imaging slice perpendicular to the main neck vessels **(A)**. The output images have a magnitude **(B)** and phase component **(C)** encoding for the flows within the bilateral internal carotid arteries (RICA and LICA), vertebral arteries (RVA and LVA) and internal jugular veins (RIJV and LIJV). **(D)** Shows a classic example of velocity aliasing artifacts on a PC-MRI phase image. This commonly happens when a too low velocity encoding (VENC) is chosen and the fast spins appear to be flowing in the opposite direction (dark cores).

For quantitative measurements, a single slice 2D PC-MRI is commonly placed on structural or angiographic MRI reference images (Figure 5A) to measure blood flow (BF) and CSF flow at the cervical level (CSFc) (Sakhare et al., 2019). This technique relies on predefined bipolar velocity encoding gradients (Battal et al., 2011) to produce reliable in-vivo imaging measurements of flow with physics details well explained in a recent work by Wymer et al. (2020). Briefly, the application of two consecutive gradients of opposing polarity results in no overall phase shifts in stationary protons, while moving protons experience a phase shift based on the distance traveled along the MRI gradient, representing their intrinsic velocity.

Two image components are therefore obtained from this flow-sensitive sequence. The magnitude component (Figure 5B) which provides structural definition of the structures where flow is observed, such as in neck arteries (bright circles), in contrast with more static tissue (dark background). The phase image component (Figure 5C), on the other hand, provides more flow-related information by encoding the spin movement in each voxel signal intensity. Importantly, depending on the direction of flow specified in the sequence parameters, and conventionally used by the manufacturer, the phase image would show high signal (bright voxels) where flow is in the same direction of the velocity encoding gradients, which are perpendicular to the imaging slice plane, (e.g., arteries in Figure 5C) and low signal (dark/black voxels) in voxels where the flow is not zero, but it is high in the opposite direction (e.g., veins in Figure 5C).

Accurate specification of velocity encoding (VENC) is crucial to avoid flow artifacts like velocity aliasing (Figure 5D), when the VENC is set too low compared to actual flow velocity, as well as poor dynamic flow ranges observed when setting the VENC too high compared to the actual flow velocities. It is therefore fundamental to set an appropriate VENC based on the expected velocities of the flow of interests that can be approximated by established literature values. Recent advancements in PC-MRI have improved spatial or temporal resolution while maintaining these principles (Wymer et al., 2020).

### 5.2 Real time PC-MRI

Real-time phase-contrast (RT-PC) MRI is a cine gradient echo sequence enabling simultaneous measurements of blood and CSF flow at high sampling rates (Lagana et al., 2022b; Baselli et al., 2022; Yildiz et al., 2017). Unlike similar sequences, RT-PC MRI is non-gated, eliminating the need for external cardiac monitoring. However, it sacrifices spatial resolution to achieve a temporal resolution of approximately 50 2D phase contrast images per cardiac cycle. In brain MRI studies, it is commonly used to assess the impact of cardiac and thoracic pump activity on cerebral blood and CSF flow. Observations from RT-PC MRI studies deepen understanding of the tight coupling between blood and CSF flow, vital for maintaining optimal intracranial pressure and brain homeostasis, in line with the Monro-Kellie doctrine (Wilson, 2016). This sequence has been used to provide important insights on the physiological factors, such as cardiac and breathing, that might drive, or at least influence, the blood and CSF flow in and out of the cranium (Lagana et al., 2022b; Lagana et al., 2022a; Yildiz et al., 2017; Dreha-Kulaczewski et al., 2018; Kollmeier et al., 2022).

Despite providing insights into physiological factors driving cranial flow, RT-PC MRI has some limitations. Its low spatial resolution makes it prone to motion artifacts and restricts measurements to large vessels. This hinders observation of flow changes in smaller structures relevant to neuronal pathologies, such as small cerebral vessels and the cerebral aqueduct. Additionally, its use in investigating body position-related flow changes is limited due to reduced spatial resolution in upright low-field strength scanners, complicating accurate region-of-interest definition. This is mostly due to the hardware specifications of such scanners which often have limiting factors such as: field strengths below 1 tesla, reduced gradient strength, slew rate and receiver channels configurations that do not support fast imaging techniques such as parallel acquisition.

### 5.3 Cardiac gated phase contrast MRI

A cardiac gating system, such as distal pulse oximeter or electrocardiogram (ECG), guides or reconstructs PC-MRI acquisition. Depending on its use, the sequence is classified as prospectively or retrospectively gated PC-MRI. In the former, a specific point of the cardiac cycle triggers rapid 2D image acquisition, covering most of the cycle by adjusting the time delay. In the latter, referred here as ReGa PC-MRI, imaging and heart rate acquisition are continuous, allowing later data reordering based on cardiac measurements for single cardiac cycle reconstruction (Frayne and Rutt, 1993).

ReGa PC-MRI offers full cardiac cycle coverage, reducing image and cardiac-related artifacts, albeit requiring additional reconstruction processing. Widely used in clinical and research cardiovascular imaging, it measures blood velocity, direction, and flow rates critical for diagnosing conditions like valvular disorders, arterial stenosis, and congenital heart abnormalities (Gatehouse et al., 2005; Schneider et al., 2001). Gated PC-MRI, beyond cardiac applications, explores cerebrovascular dynamics, revealing blood flow patterns within the brain’s vascular network. With greater spatial resolution than RT-PC MRI, it measures flows within smaller structures like the aqueduct of Sylvius and cerebral veins in healthy subjects and patients (Spijkerman et al., 2019; Ringstad et al., 2016; Kapsalaki et al., 2012). Dual velocity encoding enables simultaneous CSF and blood flow measurements, indirectly deriving properties like intracranial pressure. It’s preferred for investigating body position-related changes in larger structures like cervical CSF canals and neck arteries. However, it may miss subject-specific arrhythmias in cardiac-triggered acquisition, complicating clinical use.

**FIGURE 6:**
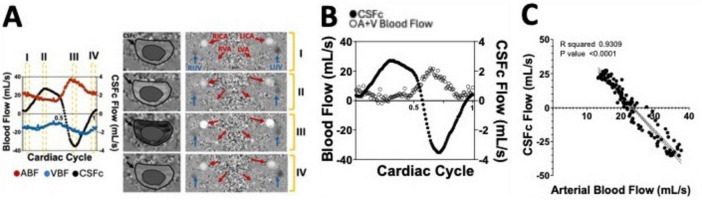
**(A)** Supine position flow measurements of arterial (ABF) and venous blood flow (VBF) as well as cervical CSF flow (CSFc), over a single cardiac cycle, extracted from a phase contrast (PC) MRI sequence whose output phase images are shown on the right. **(B)** Net blood flow calculated by adding arterial and venous (A+V) blood flow measurements over the single cardiac cycle and overlapped with the CSFc measurements. **(C)** Correlation between CSFc and arterial blood flow highlighting the strong coupling between the two neurofluids dynamics.

**FIGURE 7:**
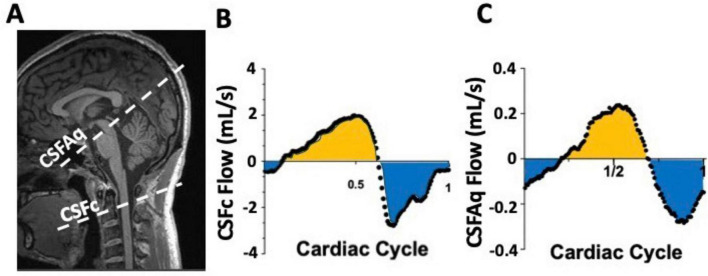
Example of phase contrast (PC) measurements of CSF flow at the cervical level (CSFc) and in the cerebral aqueduct (CSFAq) using anatomical reference image for the imaging slice placement **(A)**. Over a single cardiac cycle, notice the bidirectional nature of the CSFc flow in the cranial (yellow) and caudal (blue) direction **(B)**, mimicked by similar bidirectional flow in the aqueduct in the third ventricle (yellow) or fourth ventricle (blue) directions **(C)**.

Yet, for non-cardiac-focused studies, it normalizes acquisitions for heart rate changes, minimizing episodic influences and ensuring reliable flow quantification. Enhanced spatial resolution at higher field strengths quantifies vascular-specific properties like neurovascular compliance and vessel stiffness. This MRI approach offers a comprehensive exploration of cardiac and vascular dynamics, providing temporal and spatial insights into CNS blood and CSF flow patterns. Its sufficient spatial and temporal resolution allows relatively quick and reliable observation of major hemodynamic and hydrodynamic components, though not simultaneously acquired, reconstructed into a single cardiac cycle for overall dynamic profile analysis. Figure 6A shows how cerebral arterial and venous blood flow changes are mirrored by cervical CSF flow changes, suggesting a dynamic mechanism maintaining intracranial pressure and brain homeostasis, mainly driven by the net and arterial blood flow (Figures 6B, C). Moreover, this imaging technique has the optimal spatial and temporal properties to quantify the CSF flow at both the cervical and aqueduct level (Figures 7A–C). An important drawback of this type of sequence, however, is that the measurements will be averaged over multiple cardiac cycles. Therefore, the effects that physiological forces such as LFO and respiration have on blood and CSF flows are significantly harder to be assessed.

### 5.4 4D phase contrast

In neuroimaging, 4D PC-MRI offers a comprehensive view of brain fluid dynamics by combining 3D spatial resolution and temporal resolution of within cardiac cycle measurements. Synchronized with the cardiac cycle using ECG or pulse oximeter, it acquires flow-compensated and bidirectional flow-encoded datasets for each cardiac cycle phase (Youn and Lee, 2022; Soulat et al., 2020; Rivera-Rivera et al., 2024).

While 2D PC-MRI provides accurate velocity and flow measurements for a single slice, 4D PC-MRI sacrifices spatial resolution for broader volume coverage. This sequence applies same velocity encodings gradients as its 2D version but in all three spatial axes (x,y,z), therefore providing as output a non-flow sensitive reference image and a flow-sensitive image for each one of the axial directions (Wymer et al., 2020; Markl et al., 2012). For this reason, it excels in imaging complex structures like the circle of Willis, offering detailed visualization and quantification of flow. Recent advancements allow velocity extraction from 3D flow images, though at the expense of longer acquisition times and lower signal-to-noise ratios. This technique has recently been used to image CSF dynamics and investigate the influences that other factors such as age and morphology might play on CSF motion (Vikner et al., 2024; Malis et al., 2024).

The relatively long scanning time for this sequence introduces factors such as head motion and respiration artifacts that can significantly affect the image and data quality. In addition, the need for complex post-processing analyses combined with the harsh tradeoff between spatial resolution, signal-to-noise ratio and volume coverage are probably responsible for the relatively uncommon use of this sequence, especially compared to other techniques mentioned above. Despite these challenges, fast imaging techniques have reduced scanning times (Vikner et al., 2024; Jaeger et al., 2020) and new studies explore alternative methods for measuring body orientation-related changes in craniospinal properties, such as using head dielectric properties as indicators of craniospinal compliance changes (Vikner et al., 2020; Schubert et al., 2014; Meckel et al., 2013; Wen et al., 2019; Liu et al., 2018).

For instance, a recent study conducted by Boraschi et al. (2023) used a device to measure the head dielectric properties, composed of two electrically isolated electrodes placed on the subject’s temples, to show that, although in a small sample size of healthy young controls, body position changes including head down and head up tilts produced craniospinal compliance changes that are reflected in the dynamic changes of the head’s dielectric properties. New techniques have also been developed that allow routine MRI sequences such as functional MRI and diffusion tensor imaging (DTI) to provide details of CSF and blood flow in the human brain (Yang et al., 2022).

## 6 Neurodegeneration and aging

### 6.1 Neurofluid dynamic changes in age-related neurodegenerative diseases

Recent research suggests that CSF movement helps clear solutes from the brain along the perivascular pathways, crucial for CNS waste clearance, nutrients distribution and immune activity. Dysfunction in brain hemodynamics and hydrodynamics may contribute to neurological disorders.

For instance, altered CSF flow is seen in idiopathic normal pressure hydrocephalus (iNPH) which is commonly characterized by gait and cognitive impairments especially in the elderly, and often treated with invasive techniques such CSF shunting and lumbar drainage (Hebb and Cusimano, 2001; Wang et al., 2020; Xiao et al., 2022). INPH patients have also been shown to have greater CSF volume exchange between the third and fourth ventricles and lower CSF pulsatility at the cervical level compared to healthy individuals (Qvarlander et al., 2017). Age-related brain atrophy may also contribute to an enlargement of the overall CSF compartment (Kang et al., 2018), making iNPH a common comorbidity of other neurodegenerative diseases (Koivisto et al., 2016; Malm et al., 2013; Bech-Azeddine et al., 2007).

In diseases like Alzheimer’s (AD), Parkinson’s (PD) and multiple sclerosis (MS), disrupted brain hemodynamics and hydrodynamics are pivotal in disease progression. Impaired glymphatic system efficiency may lead to toxic protein accumulation and increased tissue cytotoxicity (Buccellato et al., 2022; Silva et al., 2021). In AD, decreased regional blood flow in areas linked to language and cognition (Leeuwis et al., 2017) correlates with disease severity and progression (Zhang et al., 2021). AD also shows impaired cerebrovascular reactivity (CVR) (Glodzik et al., 2013), reduced CSF-neuronal activity coupling (Han et al., 2021b) and impaired waste clearance through the glymphatic system (Tarasoff-Conway et al., 2015).

In PD, a decoupling between brain activity and CSF flow correlates with cognitive impairment (Han et al., 2021a; Wang et al., 2023). This aligns with reduced regional perfusion (Syrimi et al., 2017; Melzer et al., 2011), impaired blood-brain-barrier (BBB) permeability (Gray and Woulfe, 2015) and CVR (Smolinski and Czlonkowska, 2016) in PD patients.

In debilitating diseases such as MS, characterized by progressive loss of motor and cognitive skills, many brain cortical regions have shown reduced blood perfusion (Jakimovski et al., 2020; Debernard et al., 2014; Ota et al., 2013) whilst some white matter areas showed an increase in blood perfusion possibly caused by disease-related inflammation (Bester et al., 2015; Rashid et al., 2004), in line with the disease pathogenesis (Markovic-Plese and McFarland, 2001) and progression characteristics (Dobson and Giovannoni, 2019; Confavreux et al., 2000). Other studies have also reported reduced CSF stroke volume and cranial arterial blood (ElSankari et al., 2013) alongside correlation of decreased net CSF flow in the aqueduct of Sylvius with enhanced MS lesion volumes (Magnano et al., 2012).

Healthy aging also brings metabolic, vascular, and systemic changes including brain atrophy, altered brain activity (Petersen et al., 2022; MacDonald and Pike, 2021; Oschmann and Gawryluk, 2020; Kalpouzos et al., 2012), molecular and chemical changes (Gasiorowska et al., 2021; Blaszczyk, 2020). Cardiovascular changes (Strait and Lakatta, 2012) in the elderly include reduced heart rate (Fleg et al., 2005), hypertension (Buford, 2016), altered vascular morphology (Sun et al., 2022; Sun et al., 2023; Lee and Oh, 2010) and flow (Liu et al., 2012), all contributing to brain fluid dynamic changes, brain atrophy and ventricles enlargement (Vemuri et al., 2010).

Targeting brain changes from fluid dynamic alterations may offer new therapies for neurodegenerative diseases, aiming to restore waste clearance and promote brain health. For instance, new interventional techniques have showed that external stimuli can effectively alter neuronal activity (Ke et al., 2023; Williams et al., 2010; Bolognini et al., 2009), global cerebral blood flow and neuronal metabolism (Muccio et al., 2022; Stagg et al., 2013; Zheng et al., 2011) as well as brain structural (Jog et al., 2023; Hirtz et al., 2018) and functional connectivity (Kim et al., 2021; Sankarasubramanian et al., 2017; Weber et al., 2014) with parallels in clinical motor and cognitive measures (Simani et al., 2022; Eilam-Stock et al., 2021; Nissim et al., 2019; Agarwal et al., 2018; Charvet et al., 2018).

### 6.2 Body position in disease and aging

Although changes in hemodynamic and hydrodynamic properties are being reported in aging and neurodegenerative diseases, little is known on their alterations due to body position change. It has recently been observed that AD patients have a greater drop in cortical oxygenation (van Beek et al., 2010) and blood pressure (Isik et al., 2022) when moving from supine to upright, affecting cerebral perfusion. In PD, altered CBF has been reported (Camargo et al., 2015) in Niehaus et al. (2002) who found that PD patients have a smaller HR increase and take longer (5 min vs 2 min) for arterial pressure to adjust when moving to upright compared to controls. This has been confirmed by other studies reporting increased arterial blood pulsatility, pressure and autoregulation during table-tilt challenges (Xing et al., 2022; Angeli et al., 2003).

The few studies on postural effects in neurodegeneration and aging focus on either the hemodynamic or hydrodynamic effects individually, ignoring their dynamic and complementary aspects. Understanding the link between postural changes and intracranial dynamics response is crucial for comprehending disease-related impairments. Future research should explore if postural-induced changes can predict treatment effectiveness, potentially informing new therapeutic guidelines.

## 7 Conclusion

**FIGURE 8:**
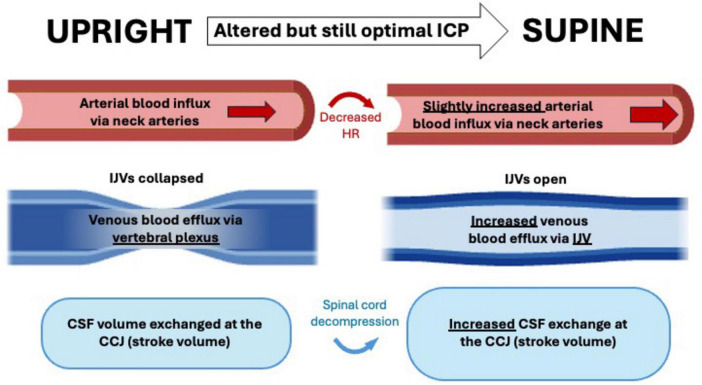
Comprehensive flowchart of the hemodynamic and hydrodynamic changes following shift in body position from upright to supine positions. Sizes of structures are not proportional and only indicative of differences between the two positions. ICP, intracranial pressure; CSF, cerebrospinal fluid; IJV, internal jugular vein; CCJ, cranio-cervical junction.

Recent neurofluid research is trending toward investigating hemodynamic and hydrodynamic changes as indicators of neurodegenerative diseases. The brain constantly adapts to varying external stimuli, making it crucial to understand how dynamic properties respond to challenges like body position shifts. Figure 8 summarizes the important findings on the effects of body position on intracranial fluid dynamics, highlighting their potential impact on a number of CNS diseases. Comparisons between body position and sleep-induced changes in intracranial blood and CSF flow are lacking, presenting an opportunity for more systematic studies. Investigating how the brain responds to daily and repetitive tasks like body position shifts will help to improve our understanding of the effects of neurofluid movement on aging and neurodegenerative processes (Smolinski and Czlonkowska, 2016; Rasmussen et al., 2018; Keage et al., 2012).

The present review offers insights into how external factors, particularly body position, influence neurofluid circulation. Despite recent advancements, research in this area remains in its early stages, necessitating further investigation, especially in the effects of body position in aging and neurodegenerative disorders.

While existing imaging techniques have provided valuable insights, improvements in spatial resolution and flow measurement accuracy are needed. PC-MRI measurements often rely on precise velocity encoding determination, requiring multiple sequences and extending scanning time. Recent advancements in artificial intelligence, machine learning, and radiomics offer promising solutions to these limitations by better detecting flow mechanics and predicting disease progression.
